# The importance of direct exchange in Kitaev magnetism

**DOI:** 10.1093/pnasnexus/pgag056

**Published:** 2026-03-03

**Authors:** Pritam Bhattacharyya, Nikolay A Bogdanov, Liviu Hozoi

**Affiliations:** Institute for Theoretical Solid State Physics, Leibniz IFW Dresden, Helmholtzstraße 20, 01069 Dresden, Germany; Department of Physics, Karpagam Academy of Higher Education, Coimbatore 641021, Tamil Nadu, India; Centre for Computational Physics, Karpagam Academy of Higher Education, Coimbatore 641021, Tamil Nadu, India; Max Planck Institute for Solid State Research, Heisenbergstraße 1, 70569, Stuttgart, Germany; Institute for Theoretical Solid State Physics, Leibniz IFW Dresden, Helmholtzstraße 20, 01069 Dresden, Germany

**Keywords:** strongly correlated materials, Kitaev–Heisenberg model, magnetic exchange

## Abstract

In magnetism research, Kitaev’s honeycomb spin model is one of the revelations of the 21st century: it hosts a quantum spin-liquid ground state that can be described analytically. Current scenarios for the occurrence of bond-dependent magnetic interactions as proposed by Kitaev rely on *indirect* exchange mechanisms based on intersite electron hopping. Analyzing the wavefunctions of Kitaev magnetic bonds at both single- and multiconfiguration levels, we find however that *direct*, Coulomb exchange may be at least as important in 5d and 4d  $t_{2g}^{5}$, 3d  $t_{2g}^{5}e_{g}^{2}$, and even rare-earth $4f^{1}$ Kitaev–Heisenberg magnets. The Coulomb exchange mechanism hints at Kitaev physics in materials that so far were not expected to host it, new ways of tuning the relative strength of the Kitaev interaction, and new design principles for Kitaev spin liquids.

Significance Statement Computational data at the most basic level of approximation in ab initio electronic structure theory, Hartree–Fock-like, point to a new ingredient in Kitaev magnetism: orbital-dependent *direct* exchange. In 3*d* and 5*d* Kitaev–Heisenberg magnets it even defines the leading intersite interaction. This ab initio radiography of the interaction landscape not only uncovers a new element in the plot, it also reveals the limits of standard computational approaches, model-Hamiltonian and/or density-functional-based, to electronic structures and interaction mechanisms in magnetic insulators.

A magnet is a collection of magnetic moments. How those interact is determined by what lies in between. In transition-metal and rare-earth magnetic compounds, the configuration of the ligands around each magnetic center and the connectivity of the ligand cages are therefore pivotal—for example, the mutual interaction of magnetic species connected through one single ligand is qualitatively different from the case of two bridging anions. The glue of this interaction is exchange.

*Direct* exchange occurs through the interplay of Pauli’s exclusion principle and Coulomb repulsion, as discussed by Heisenberg, Dirac, and van Vleck already in the 1920s (1–3), has no classical analog, and is the main effect responsible for ferromagnetism. The antiferromagnetic ground states observed in a variety of magnetic insulators, on the other hand, arise from *indirect* exchange interactions involving intersite electron hopping: M–M kinetic exchange, where only electrons at the magnetic centers are active, and M–L–M superexchange, where electrons at nonmagnetic, intermediary ionic sites (eg chalcogenide or halide ligands) are also relocated.

For valence-band effective models with one, half-filled orbital per magnetic ion and $180^{\circ }$ M–L–M chemical bonds, kinetic exchange and superexchange imply rather simple analytical expressions. Such physics took center stage in studies of the copper-oxide superconductors (4), leaving direct exchange in the shade. More recently, kinetic exchange and superexchange were discussed in the context of anisotropic Kitaev interactions (5) on networks of edge-sharing $t_{2g}^{5}$ (6–9), $t_{2g}^{5}e_{g}^{2}$ (10–13), and $4f^{1}$ (14, 15) ${\mathrm{ML}}_{6}$ octahedra. However, different from the case of corner-sharing ${\mathrm{ML}}_{6}$ octahedra and $180^{\circ }$ M–L–M links in superconducting cuprates (where the direct M–M orbital overlap is small), for edge-sharing ${\mathrm{ML}}_{6}$ units and $90^{\circ }$ (or $\approx 90^{\circ }$) M–L–M paths (6–15), direct exchange may in principle become comparable in size with the indirect exchange mechanisms, especially for M-site orbitals with lobes along the M–M axis. Yet, direct exchange has been completely ignored so far in phenomenological Kitaev–Heisenberg exchange models (6–15).

Even for corner-sharing ${\mathrm{ML}}_{n}$ units, there are situations where direct exchange may again compete with the indirect, hopping-mediated exchange mechanisms: strongly bent M–L–M paths, especially in the cases of adjacent pyramidal ${\mathrm{ML}}_{5}$ entities, adjacent ${\mathrm{ML}}_{4}$ tetrahedra, and mixed types of polyhedra, networks of corner-sharing ${\mathrm{ML}}_{6}$ octahedra and ${\mathrm{ML}}_{4}$ tetrahedra. Mingled polyhedra—in particular, octahedra and tetrahedra—are encountered in some of the most promising multiferroic/magnetoelectric materials, ie the Y-type hexaferrites (16), and in the family of ${\mathrm{Fe}}_{2}{\mathrm{Mo}}_{3}O_{8}$ (17) and ${\mathrm{Co}}_{2}{\mathrm{Mo}}_{3}O_{8}$ (18) multiferroics.

How direct and indirect exchanges work for Kitaev–Heisenberg magnetic centers that cover the whole correlated-electron sector of the periodic table is illustrated at ab initio level in the following, by means of wavefunction electronic-structure theory (19, 20).

## Results

### The $A_{3}{BM}_{2}L_{6}$ material platform, $t_{2g}^{5}$ vs. $t_{2g}^{5}e_{g}^{2}$ Kitaev centers

A basic ingredient for exotic magnetic ground states and responses is frustration. Typical geometrically frustrated magnetic lattices are the triangular, kagomé, and pyrochlore networks. The triangular set-up is the simplest. It is encountered in eg rhombohedral crystalline arrangements derived from the rocksalt structure, in the form of successive sheets of edge-sharing ${\mathrm{ML}}_{6}$ octahedra perpendicular to the 〈111〉 direction (see, eg discussion in Ref. (21)). Hexagonal architectures can be obtained out of the triangular layers if certain magnetic sites are removed or occupied by nonmagnetic atomic species (7, 21, 22). In hexagonal setting, frustration may occur only through anisotropic effective coupling constants, diagonal (ie Kitaev) or/and off-diagonal. Many triangular and hexagonal magnets can be generically described through the chemical formula $A_{3}{\mathrm{BM}}_{2}L_{6}$ (sometimes written as $A_{3}M_{2}{\mathrm{BL}}_{6}$) (22). For example, B can be Li in the spin-liquid honeycomb iridate $H_{3}{\mathrm{LiIr}}_{2}O_{6}$ (23) or Sb in the cobaltates ${\mathrm{Li}}_{3}{\mathrm{Co}}_{2}{\mathrm{SbO}}_{6}$ (24) and ${\mathrm{Na}}_{3}{\mathrm{Co}}_{2}{\mathrm{SbO}}_{6}$ (25); A=B=Na, M=Ir, and L=O gives ${\mathrm{Na}}_{2}{\mathrm{IrO}}_{3}$, a representative 5d Kitaev–Heisenberg honeycomb magnet (9); A=B=0 (ie empty A and B sites), M=Ru, and L=Cl corresponds to ${\mathrm{RuCl}}_{3}$, a 4d Kitaev–Heisenberg honeycomb system (9); with B=M we arrive to ${\mathrm{AML}}_{2}$ delafossite-type triangular structures, eg ${\mathrm{NaRuO}}_{2}$ (26, 27), ${\mathrm{CoI}}_{2}$ (with unoccupied A sites) (28), and ${\mathrm{RbCeO}}_{2}$ (29); A=B=M=Co and L=O corresponds to rocksalt CoO (ie successive triangular Co-ion and O layers normal to the 〈111〉 direction).

For the case of edge-sharing ${\mathrm{ML}}_{6}$ octahedra with $t_{2g}^{5}$ valence electron configuration at the magnetic sites, the interplay of $t_{2g}$-shell spin–orbit coupling, intersite hopping, and on-site (Hund) exchange were shown to generate anisotropic exchange à la Kitaev (5) (*indirect*, hopping mediated) already 15 years ago (7). However, the *direct*, Coulomb M–M exchange amplitudes should also be sizable, especially those implying *σ*- and *π*-type pairs of orbitals—the interplay between such orbital-dependent Coulomb exchange and $t_{2g}$-shell spin–orbit coupling is another possible source of anisotropic magnetism. The roles of the different mechanisms can be easily verified with ab initio wavefunction electronic structure computational methods (19). Such calculations have been used for a long time to explore solid-state electronic structures and can provide information that is not accessible by other means, on eg nontrivial correlated wavefunctions (30, 31), cohesive energies (32), band gaps (33, 34), and, of particular interest here, exchange mechanisms (35–37).

**Fig. 1.**
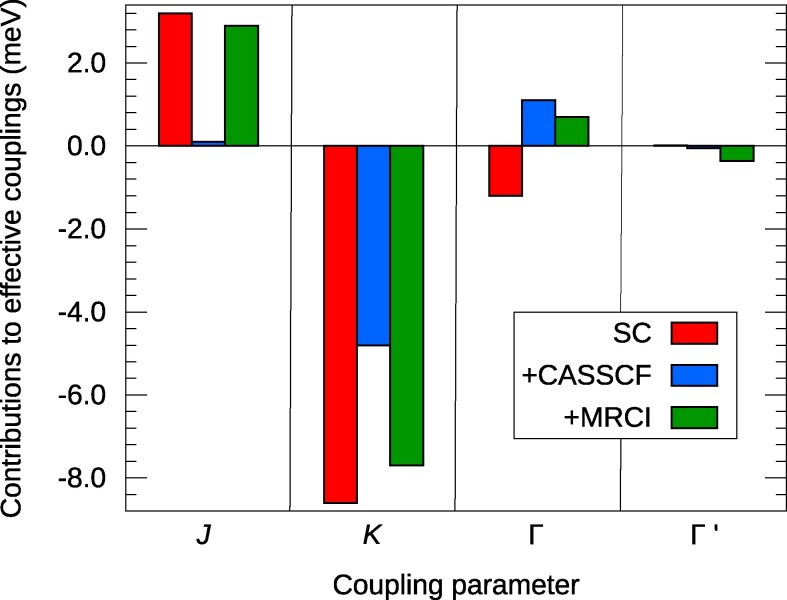
Exchange contributions to the intersite magnetic couplings in $5d^{5}$  ${\mathrm{Na}}_{2}{\mathrm{IrO}}_{3}$: Coulomb exchange (SC results, in red), $\mathrm{Ir}(t_{2g})$–$\mathrm{Ir}(t_{2g})$ kinetic exchange (as the difference between CASSCF and SC data, in blue), plus contributions related to Ir-$O_{2}$-Ir superexchange, $\mathrm{Ir}(t_{2g})\to \mathrm{Ir}(e_{g})$ excitations, and so called dynamical correlation effects (19) (as the difference between MRCI and CASSCF, in green).

**Fig. 2.**
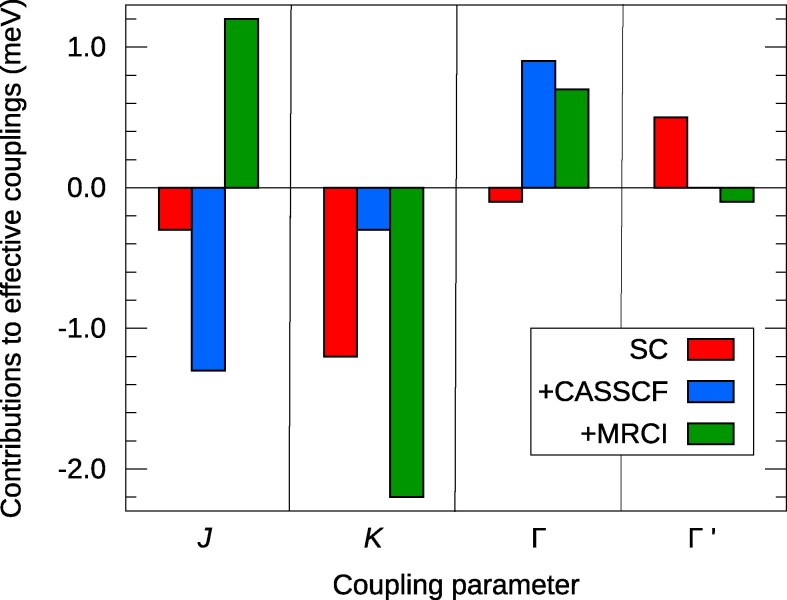
Contributions to the intersite magnetic couplings in $4d^{5}$  ${\mathrm{RuCl}}_{3}$: Coulomb exchange (red bars), $\mathrm{Ru}(t_{2g})$–$\mathrm{Ru}(t_{2g})$ kinetic exchange (blue), plus contributions related to Ru-${\mathrm{Cl}}_{2}$-Ru superexchange, $\mathrm{Ru}(t_{2g})\to \mathrm{Ru}(e_{g})$ excitations, and dynamical correlation (green).

Focusing first on the hitherto neglected direct exchange mechanism, spin–orbit calculations that account only for the leading $t_{2g}^{5}$–$t_{2g}^{5}$ ground-state electron configuration (to which we refer as single-configuration, SC, computations) and subsequent mapping (38) onto the effective nearest-neighbor spin Hamiltonian (see Materials and methods for further details) indicate indeed large contributions. Those are shown as red bars in Figs. 1 and 2, for ${\mathrm{Na}}_{2}{\mathrm{IrO}}_{3}$ and ${\mathrm{RuCl}}_{3}$, prototype $t_{2g}^{5}$ Kitaev–Heisenberg honeycomb magnets (7–9). Besides the isotropic Heisenberg *J* and diagonal anisotropic *K* couplings, the off-diagonal Γ and $\Gamma^{\prime }$ effective coupling parameters are analyzed as well in the two figures. They enter the effective Hamiltonian for a pair of adjacent 1/2-pseudospins ${\tilde{S}}_{i}$ and ${\tilde{S}}_{j}$ as (8, 9)


(1) $$H_{ij}^{\left(\gamma \right)}=J{\;\tilde{S}}_{i}\cdot {\;\tilde{S}}_{j}+K{\tilde{S}}_{i}^{\gamma }{\tilde{S}}_{j}^{\gamma }+\sum_{\alpha \neq \beta }\Gamma_{\alpha \beta }({\tilde{S}}_{i}^{\alpha }{\tilde{S}}_{j}^{\beta }+{\tilde{S}}_{i}^{\beta }{\tilde{S}}_{j}^{\alpha }),$$

with α,β,γ∈{x,y,z}. For eg a *z*-type M–M bond (ie $M_{2}L_{2}$ plaquette normal to the *z* axis), $\Gamma \equiv \Gamma_{xy}$ and $\Gamma^{\prime }\equiv \Gamma_{yz}=\Gamma_{zx}$.

The indirect mechanisms, kinetic exchange (blue) and superexchange (green), require more involved computations, multiconfiguration complete-active-space self-consistent-field (CASSCF) wavefunction expansions (19, 39) that account for intersite excitations within the transition-ion $t_{2g}$ sector and multireference configuration-interaction (MRCI) wavefunctions (19, 40) including also L-to-M excitations, respectively (see Materials and methods for computational details). Remarkably, direct exchange brings the largest contributions to *K*, *J*, and Γ in 5d  ${\mathrm{Na}}_{2}{\mathrm{IrO}}_{3}$ (as shown in Fig. 1) and to $\Gamma^{\prime }$ in 4d  ${\mathrm{RuCl}}_{3}$ (Fig. 2). It also provides sizable weight to the Kitaev coupling *K* in ${\mathrm{RuCl}}_{3}$, ≈33%.

**Fig. 3.**
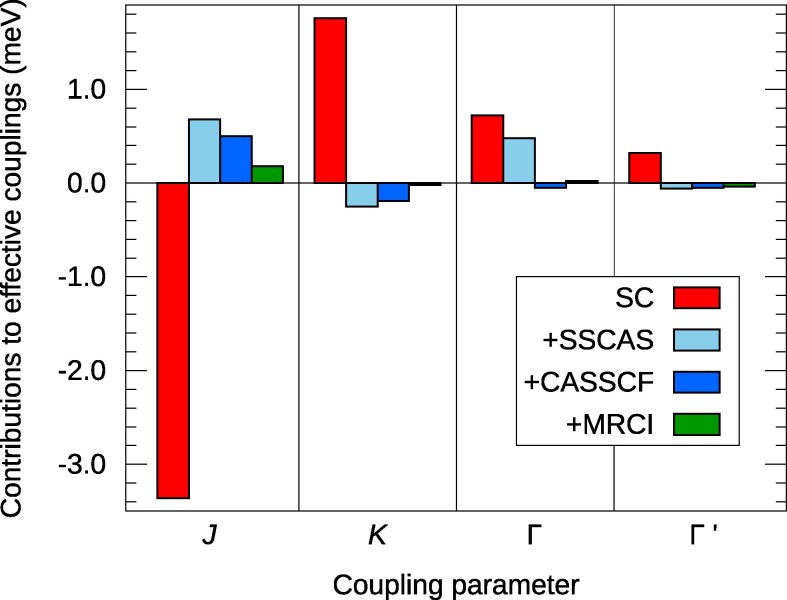
Exchange mechanisms contributing to the intersite magnetic couplings in $3d^{7}$  ${\mathrm{Li}}_{3}{\mathrm{Co}}_{2}{\mathrm{SbO}}_{6}$: *d–d* Coulomb exchange (red bars), renormalization due to on-site intra-3d excitations (light blue), *d–d* kinetic exchange (dark blue), plus contributions related to Co–$O_{2}$–Co superexchange and dynamical correlation (green).

The role of direct exchange is even more spectacular in the case of $t_{2g}^{5}e_{g}^{2}$  3d compounds, eg ${\mathrm{Li}}_{3}{\mathrm{Co}}_{2}{\mathrm{SbO}}_{6}$: direct exchange is the dominant exchange mechanism for all four effective parameters, as illustrated in Fig. 3. To clearly identify the role of kinetic exchange, two different sets of multiconfiguration calculations were performed: first accounting only for on-site intra-3d excitations, referred to as single-site complete-active-space (SSCAS, with contributions depicted in light blue in Fig. 3) and then for all possible intra-3d excitations, both on-site and intersite (with additional contributions shown in darker blue). Renormalization effects as found in the SSCAS computation, through second-order spin–orbit interactions, might also play a significant role in $3d^{8}$ nickelates such as ${\mathrm{NiI}}_{2}$ (41). The numerical values obtained at different levels of approximation are provided in Table 1.

**Table 1.** Effective magnetic couplings at different levels of approximation for $C_{2h}$  $M_{2}L_{10}$ two-octahedra units in the Kitaev–Heisenberg systems ${\mathrm{Li}}_{3}{\mathrm{Co}}_{2}{\mathrm{SbO}}_{6}$ (24), ${\mathrm{RuCl}}_{3}$ (42), ${\mathrm{Na}}_{2}{\mathrm{IrO}}_{3}$ (43), and ${\mathrm{RbCeO}}_{2}$ (29).

	*J*	*K*	Γ	$\Gamma^{\prime }$
3*d* ${\mathrm{Li}}_{3}{\mathrm{Co}}_{2}{\mathrm{SbO}}_{6}$ (meV)				
SC	−3.4	1.8	0.7	0.3
SSCAS	−2.7	1.5	1.2	0.3
CASSCF	−2.2	1.3	1.1	0.2
MRCI	−2.0	1.3	1.2	0.2
4*d* ${\mathrm{RuCl}}_{3}$ (meV)				
SC	−0.3	−1.2	−0.1	0.5
CASSCF	−1.6	−1.5	0.8	0.5
MRCI	−0.4	−3.7	1.5	0.4
5*d* ${\mathrm{Na}}_{2}{\mathrm{IrO}}_{3}$ (meV)				
SC	3.2	−8.6	−1.2	0.01
CASSCF	3.3	−13.4	−0.1	−0.04
MRCI	6.2	−21.1	0.6	−0.4
4*f* ${\mathrm{RbCeO}}_{2}$ (μeV)				
SSCAS	−10.3	−37.3	−9.1	−7.1
CASSCF	59.4	−28.3	−8.8	−5.4

### $4f^{1}$ –$4f^{1}$ anisotropic direct exchange

Recently, quantum spin liquid (QSL) behavior has been reported in a number of triangular-lattice pseudospin-1/2 $4f^{13}$ and $4f^{1}$ chalcogenides: ${\mathrm{YbMgGaO}}_{4}$ (44), ${\mathrm{NaYbS}}_{2}$ (45), ${\mathrm{NaYbO}}_{2}$ (46), ${\mathrm{NaYbSe}}_{2}$ (47), ${\mathrm{CsYbSe}}_{2}$ (48), ${\mathrm{KYbSe}}_{2}$ (49), ${\mathrm{RbYbSe}}_{2}$ (49), and ${\mathrm{RbCeO}}_{2}$ (29). Given the smaller (or comparable (29)) energy scale of the 4f crystal-field splittings with respect to the strength of the spin–orbit coupling *λ*, there are 7×7=49 configurations that must be explicitly considered in the spin–orbit treatment for $4f^{1}$–$4f^{1}$ and 4$f^{13}$–$4f^{13}$ pairs of ions. The single-site ground-state Kramers doublet is typically separated from the lowest on-site excitations by a sizable gap; when mapping the ab initio data onto the effective two-site magnetic model, considering only the lowest four “magnetic” states out of the whole set of 196 is then a good approximation. The model-Hamiltonian studies on $4f^{1}$–$4f^{1}$ and $4f^{13}$–$4f^{13}$ (super)exchange are also performed along this idea (14, 15, 50, 51).

**Fig. 4.**
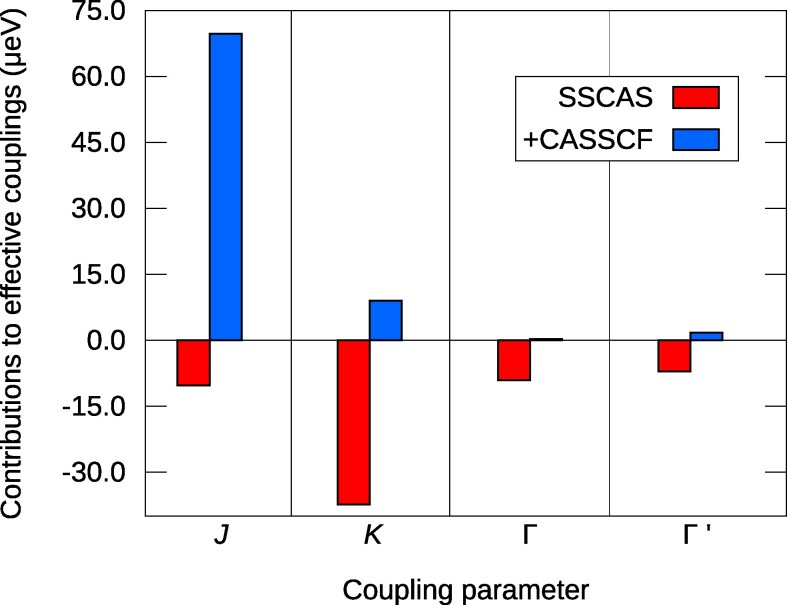
4f –4f Coulomb exchange (red) and 4f–4f kinetic exchange (blue) in ${\mathrm{RbCeO}}_{2}$.

Mapping the lowest four eigenstates obtained by spin–orbit 4f SSCAS and 4f CASSCF two-octahedra computations onto the effective magnetic Hamiltonian described by (1), it was possible to estimate the role of direct and kinetic exchange, respectively, for the effective intersite couplings in $4f^{1}$  ${\mathrm{RbCeO}}_{2}$ (Fig. 4), a triangular-lattice rare-earth system that does not order magnetically down to 60 mK (29). It is found that for the anisotropic channel (*K*, Γ, and $\Gamma^{\prime }$) the direct exchange contributions are very important (see also the data in Table 1), larger than what kinetic exchange brings. Spin–orbit MRCI computations for two adjacent ${\mathrm{CeO}}_{6}$ octahedra (to estimate Ce–$O_{2}$–Ce superexchange contributions) are computationally quite demanding and will constitute the topic of a different study.

## Discussion

A 21st-century revelation in magnetism research is Kitaev’s honeycomb-lattice anisotropic spin model, in particular, the seemingly counterintuitive directional dependence of its anisotropic intersite couplings, the peculiar flavor of QSL ground state that the model hosts, and the possibility of describing the QSL analytically (5). With Khaliullin’s and Jackeli’s remarkable intuition and pioneering work (6, 7), we know how anisotropic (pseudo)spin interactions à la Kitaev may arise in quantum matter and in which kind of magnets we should look for those. However, it appears that the Kitaev (pseudo)spin interaction tableau is not yet fully uncovered: through ab initio, wavefunction computations here we reveal an additional Kitaev interaction mechanism—direct, Coulomb exchange (also referred to as potential exchange) in the presence of sizable spin–orbit coupling. It turns out that in prototype Kitaev–Heisenberg magnets such as ${\mathrm{Na}}_{2}{\mathrm{IrO}}_{3}$ and ${\mathrm{Li}}_{3}{\mathrm{Co}}_{2}{\mathrm{SbO}}_{6}$ it actually represents the leading intersite interaction. Moreover, it seemingly brings important contributions to the anisotropic interactions on 4f-ion triangular lattices.

The massive Coulomb exchange contributions reported here represent very solid data, all those are obtained at the lowest possible level of approximation in ab initio electronic-structure theory, Hartree–Fock-like. Similar results on the magnitude of the intersite Coulomb exchange contributions should be obtained by density-functional computations using functionals that build in exact (ie Hartree–Fock) exchange and completely disregard correlations.^a^

Direct, Coulomb exchange adds an extra dimension to the Kitaev–Heisenberg interaction landscape. An important aspect that needs to be understood is the interplay of direct and indirect exchange mechanisms, eg how those different contributions can be tuned to 0 in the case of the Heisenberg *J*, such that the Kitaev QSL phase is stabilized. This would provide theoretical guidelines to eg experiments under strain on Kitaev–Heisenberg magnets. That the different exchange mechanisms may compete with each other is apparent in Fig. 2, for the isotropic component in ${\mathrm{RuCl}}_{3}$: direct and kinetic exchange (red and blue bars) compete with and are nearly counterbalanced by superexchange and additional correlation effects accounted for in MRCI (green). It is worth noting that the sum of the different effects in the isotropic channel agrees with the small *J* value derived from eg neutron scattering measurements on ${\mathrm{RuCl}}_{3}$ (52). The analysis vs. experimental data is also illustrative for the case of the $A_{3}{\mathrm{Co}}_{2}{\mathrm{SbO}}_{6}$ cobaltates: the *leading* Coulomb-exchange contribution—ferromagnetic, isotropic, stronger in ${\mathrm{Li}}_{3}{\mathrm{Co}}_{2}{\mathrm{SbO}}_{6}$ (−3.4 meV, see Table 1) than in ${\mathrm{Na}}_{3}{\mathrm{Co}}_{2}{\mathrm{SbO}}_{6}$ (−1.4 meV (53))—seemingly explains (i) the ferromagnetic Curie–Weiss temperatures found experimentally in these compounds (25, 54) and (ii) a Curie–Weiss temperature that is larger in ${\mathrm{Li}}_{3}{\mathrm{Co}}_{2}{\mathrm{SbO}}_{6}$ (15 K (54)) than in ${\mathrm{Na}}_{3}{\mathrm{Co}}_{2}{\mathrm{SbO}}_{6}$ (2 K (25)).

## Materials and methods

All quantum chemical computations were carried out using the Molpro suite of programs (55). For each type of embedded cluster, the crystalline environment was modeled as a large array of point charges which reproduces the crystalline Madelung field within the cluster volume; we employed the Ewald program (56) to generate the point-charge embeddings.

The many-body ab initio calculations were performed for fragments consisting of two central octahedra and either four (for hexagonal lattices) or eight (for the triangular compound) adjacent octahedra. CASSCF computations were carried out with six $t_{2g}$ orbitals and 10 electrons as active for the iridate and ruthenate systems, with the 10 valence 3d orbitals and 14 electrons in the active space for the cobaltate, and with 14 4f orbitals and two electrons for the $4f^{1}$ system. The CASSCF optimizations were performed for all possible spin multiplicities: lowest nine singlets and nine triplets associated with the leading $t_{2g}^{5}$–$t_{2g}^{5}$ configuration for the iridate and ruthenate, lowest nine singlet, nine triplet, nine quintet, and nine septet states associated with the leading $t_{2g}^{5}e_{g}^{2}$–$t_{2g}^{5}e_{g}^{2}$ ground-state configuration for the cobaltate, and lowest 49 singlets and 49 triplets associated with the $f^{1}$–$f^{1}$ configuration for ${\mathrm{RbCeO}}_{2}$.

Different from previous quantum chemical investigations (eg on ${\mathrm{RuCl}}_{3}$ in Ref. (38)), where the core and semicore orbitals were kept frozen at CASSCF level, as obtained from a preliminary Hartree–Fock calculation preceding the CASSCF step, all orbitals were here reoptimized in the CASSCF variational procedure. Interestingly, for the particular case of ${\mathrm{RuCl}}_{3}$, by full orbital optimization in CASSCF the sign of the Heisenberg *J* is reversed: from J=1.2 meV in Ref. (38), we arrive at J=−0.4 meV in the final MRCI spin–orbit computation (Table 1) if all orbitals are reoptimized in CASSCF. The other nearest-neighbor coupling parameters are less affected.

In the subsequent MRCI correlation treatment, single and double excitations out of the central-unit magnetic d/f and bridging-ligand *p* orbitals were considered (for the cobaltate, O $2p_{z}$ only). Spin–orbit couplings were further accounted for as described in Ref. (57), either at SC, SSCAS, CASSCF, or MRCI level. The lowest four spin–orbit eigenstates from the Molpro output (with eigenvalues lower by ∼30 meV or more compared to other states) were mapped onto the eigenvectors of the effective spin Hamiltonian (1), following the procedure described in Refs. (38, 58).

We used the Pipek–Mezey methodology (59) to obtain localized central-unit orbitals. The localized orbitals (LOs) allow to construct SC wavefunctions (using appropriate restrictions in the Molpro inputs for the occupations of the LOs) and subsequently derive the Coulomb exchange contributions to the effective nearest-neighbor magnetic couplings (ie the red bars in Figs. 1–4). Illustrative LO plots and information concerning the atomic basis sets are provided in Supporting Information. Orbital composition analysis through Mulliken partition (60, 61) yields 99% Co 3d character for the Co $t_{2g}$ LOs and 97% Co 3d character for the Co $e_{g}$ LOs in ${\mathrm{Li}}_{3}{\mathrm{Co}}_{2}{\mathrm{SbO}}_{6}$, 94% Ru 4d character for the Ru $t_{2g}$ magnetic LOs in ${\mathrm{RuCl}}_{3}$, 90% Ir 5d character for the Ir $t_{2g}$ magnetic LOs in ${\mathrm{Na}}_{2}{\mathrm{IrO}}_{3}$, and 99.5% Ce 4f character for the magnetic LOs in ${\mathrm{RbCeO}}_{2}$. No orbital optimization was further performed in the SC and SSCAS computations; the latter can be described as occupation-restricted multiple active space CI calculations (62).

Lattice parameters as determined in Refs. (24, 29, 42, 43) were respectively employed for ${\mathrm{Na}}_{2}{\mathrm{IrO}}_{3}$, *α*-${\mathrm{RuCl}}_{3}$, ${\mathrm{Li}}_{3}{\mathrm{Co}}_{2}{\mathrm{SbO}}_{6}$, and ${\mathrm{RbCeO}}_{2}$.

## Supplementary Material

pgag056_Supplementary_Data

## Data Availability

Data used in the study are included in the manuscript and/or supporting information.

## Notes

a On the other hand, describing kinetic exchange and superexchange through the exchange-correlation functional remains elusive.
