# Supplementary material for: The importance of direct exchange in Kitaev magnetism
Source: PNAS Nexus. 2026 Mar 3;5(3):pgag056. doi: 10.1093/pnasnexus/pgag056 (PMC13016923; doi:10.1093/pnasnexus/pgag056)
Supplement: pgag056_Supplementary_Data [file pgag056_supplementary_data.pdf]

Supporting Information — The importance of direct exchange in Kitaev magnetism

Pritam Bhattacharyya,[1,2,3] Nikolay A. Bogdanov,[4] and Liviu Hozoi[1]

[1] *Institute for Theoretical Solid State Physics, Leibniz IFW Dresden, Helmholtzstraße 20, 01069 Dresden, Germany*
[2] *Department of Physics, Karpagam Academy of Higher Education, Coimbatore 641021, Tamil Nadu, India*
[3] *Centre for Computational Physics, Karpagam Academy of Higher Education, Coimbatore 641021, Tamil Nadu, India*
[4] *Max Planck Institute for Solid State Research, Heisenbergstraße 1, 70569 Stuttgart, Germany*

(Dated: December 28, 2025)

Basis set information

Na_2IrO_3. Relativistic pseudopotentials (ECP60MDF) and basis sets (BSs) of effective quadruple-ζ quality (ECP60MDF-VTZ) [1] were utilized for the two 'central' Ir ions. All-electron BSs of quintuple-ζ quality were employed for the two bridging ligands [2] while all-electron triple-ζ BSs were applied for the remaining eight O anions [2] associated with the two octahedra of the reference magnetic unit. The four adjacent transition ions were represented as closed-shell Pt^{4+} t_{2g}^6 species, using relativistic pseudopotentials (Ir ECP61MDF) and (Ir ECP60MDF-VDZ) (8s7p6d)/[3s3p3d] BSs [1]; the t_{2g} orbitals of these adjacent cations were part of the inactive orbital space. The other 16 O ligands associated with the four adjacent transition metal sites were described through minimal all-electron atomic natural orbital (ANO) BSs [3]. Large-core pseudopotentials were employed for the 18 Na nearest neighbors [4].

α-$RuCl_3$. We employed energy-consistent relativistic pseudopotentials (ECP28MDF) and Gaussian-type valence BSs of effective quadruple-ζ quality (ECP28MDF-VTZ) [5] for the central Ru species. All-electron BSs of quintuple-ζ quality were utilized for the two bridging ligands [6] and of triple-ζ quality for the remaining eight Cl anions [6] linked to the two octahedra of the reference unit. The four adjacent cations were represented as closed-shell Rh^{3+} t_{2g}^6 species, using relativistic pseudopotentials (Ru ECP29MDF) and (Ru ECP28MDF-VDZ) (8s7p6d)/[3s3p3d] BSs for electrons in the 4th shell [5]; the outer 16 Cl ligands associated with the four adjacent octahedra were described through minimal ANO BSs [3].

$Li_3Co_2SbO_6$. We utilized all-electron BSs of quadruple-ζ quality for the central Co sites, [7s6p4d2f] [7]. All-electron BSs of quintuple-ζ quality were employed for the two bridging ligands [2] while all-electron triple-ζ BSs were applied for the remaining eight O anions [2] associated with the two octahedra of the reference unit. The four adjacent transition ions were represented as closed-shell Zn^{2+} cations, using large-core pseudopotentials Zn ECP28MWB plus uncontracted (3s2p) valence BSs [8]), and the four adjacent Sb species through large-core pseudopotentials Sb ECP46MDF plus (4s4p)/[2s2p] valence BSs [9]. The outer 14 O ligands associated with the four adjacent SbO_6 octahedra were described through minimal all-electron ANO BSs [3]. Large-core pseudopotentials were considered for the 24 Li nearby cations [4].

$RbCeO_2$. We used ECP28MWB quasirelativistic pseudopotentials [10] and Gaussian ANO valence BSs [11, 12] for the central Ce species. All-electron BSs of quintuple-ζ quality were utilized for the two bridging ligands [13] and of triple-ζ quality for the remaining eight O anions [13] of the two octahedra of the reference magnetic unit. For the eight Ce neighbors, we employed large-core quasirelativistic pseudopotentials (ECP47MWB) [14, 15]. Large-core pseudopotentials were also considered for the 18 Rb nearby cations [16, 17].

Orbital basis for computing exchange contributions

The analysis of exchange contributions was carried out in terms of localized central-unit orbitals obtained through Pipek-Mezey localization [18]. The single-configuration (SC) wavefunctions were constructed using appropriate restrictions for the occupations of the localized orbitals (LOs), such that intersite excitations are excluded. From orbital composition analysis through Mul-

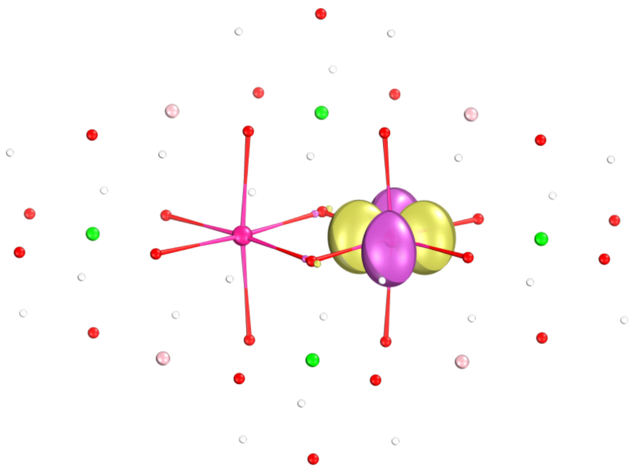

FIG. S1. Localized Co 3d xy magnetic orbital in $Li_3Co_2SbO_6$, plot with 95% of the electron density within the contour; for plots with less than 94% of the electron density within the contour, the O p tails are not at all visible. Bonds are depicted only for the Co_2O_{10} block of two edge-sharing octahedra; other atomic sites shown in the figure define the quantum mechanical cluster described in the previous section.

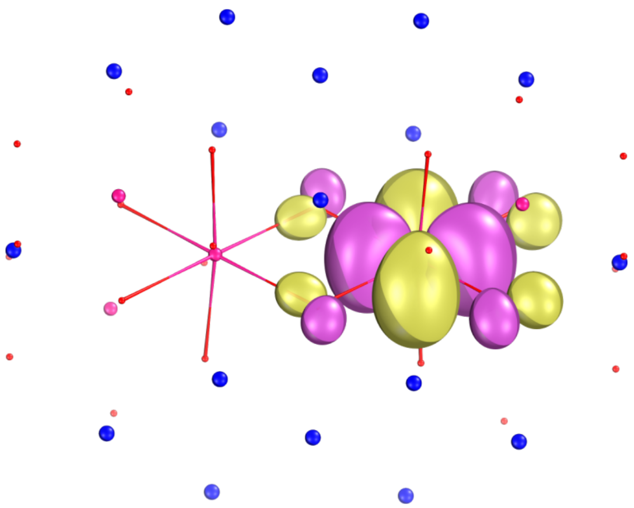

FIG. S2. Localized Ir 5d xy magnetic orbital in Na_2IrO_3, plot with 90% of the electron density within the contour. For comparison, a localized O 2p valence orbital is depicted in Fig. S3.

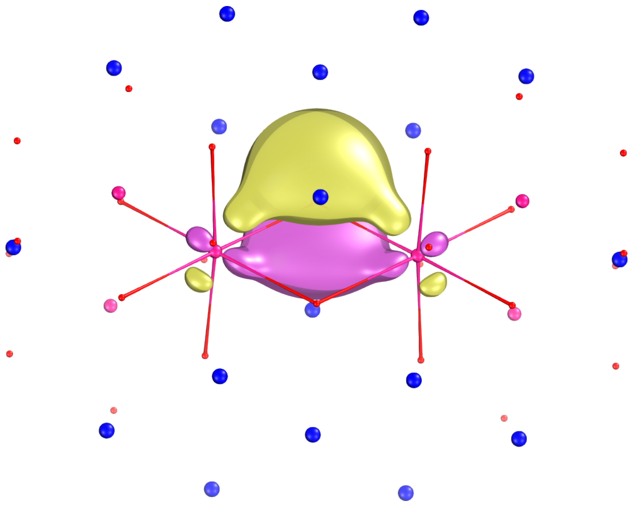

FIG. S3. Localized O 2p orbital in Na_2IrO_3, plot with 90% of the electron density within the contour.

liken partition [19, 20], the tails at adjacent sites of the magnetic LOs are $\lesssim$1% in $RbCeO_2$, $\lesssim$3% in $Li_3Co_2SbO_6$, $\approx$ 6% in $RuCl_3$, and $\approx$ 10% in Na_2IrO_3. Illustrative LO plots are provided for $Li_3Co_2SbO_6$ and Na_2IrO_3 in Figs. S1-S3; the visualization program IboView [21] was employed.

---

[1] D. Figgen, K. A. Peterson, M. Dolg, and H. Stoll, J. Chem. Phys. **130**, 164108 (2009).

[2] T. H. Dunning, J. Chem. Phys. **90**, 1007 (1989).

[3] K. Pierloot, B. Dumez, P.-O. Widmark, and B. O. Roos, Theor. Chim. Acta **90**, 87 (1995).

[4] P. Fuentealba, H. Preuss, H. Stoll, and L. Von Szentpály, Chem. Phys. Lett. **89**, 418 (1982).

[5] K. A. Peterson, D. Figgen, M. Dolg, and H. Stoll, J. Chem. Phys. **126**, 124101 (2007).

[6] D. E. Woon and T. H. Dunning Jr., J. Chem. Phys. **98**, 1358 (1993).

[7] N. B. Balabanov and K. A. Peterson, J. Chem. Phys. **123**, 064107 (2005).

[8] F. Schautz, H.-J. Flad, and M. Dolg, Theor. Chem. Acc. **99**, 231 (1998).

[9] H. Stoll, B. Metz, and M. Dolg, J. Comput. Chem. **23**, 767 (2002).

[10] M. Dolg, H. Stoll, and H. Preuss, J. Chem. Phys. **90**, 1730 (1989).

[11] X. Cao and M. Dolg, J. Chem. Phys. **115**, 7348 (2001).

[12] X. Cao and M. Dolg, J. Mol. Struct. THEOCHEM **581**, 139 (2002).

[13] J. Dunning, Thom H., J. Chem. Phys. **90**, 1007 (1989).

[14] M. Dolg, H. Stoll, A. Savin, and H. Preuss, Theor. Chim. Acta **75**, 173 (1989).

[15] M. Dolg, H. Stoll, and H. Preuss, Theor. Chim. Acta **85**, 441 (1993).

[16] L. von Szentpály, P. Fuentealba, H. Preuss, and H. Stoll, Chem. Phys. Lett. **93**, 555 (1982).

[17] P. Fuentealba, H. Stoll, L. von Szentpály, P. Schwerdtfeger, and H. Preuss, J. Phys. B: Atom. Mol. Phys. **16**, L323 (1983).

[18] J. Pipek and P. G. Mezey, J. Chem. Phys. **90**, 4916 (1989).

[19] T. Lu and Q. Chen, *Exploring Chemical Concepts Through Theory and Computation* (John Wiley & Sons, 2024) Chap. 6, pp. 161–188.

[20] T. Lu, J. Chem. Phys. **161**, 082503 (2024).

[21] G. Knizia, J. Chem. Theory Comput. **9**, 4834 (2013).
